# Erythropoietin in bone homeostasis—Implications for efficacious anemia therapy

**DOI:** 10.1002/sctm.20-0387

**Published:** 2021-01-21

**Authors:** Katrina M. Lappin, Ken I. Mills, Terence R. Lappin

**Affiliations:** ^1^ Patrick G Johnston Centre for Cancer Research Queen's University Belfast Belfast UK

**Keywords:** bone, erythropoietin, hematopoiesis, osteoblast

## Abstract

Bone homeostasis and hematopoiesis are irrevocably linked in the hypoxic environment of the bone marrow. Erythropoietin (Epo) regulates erythropoiesis by binding to its receptor, Epor, on erythroid progenitor cells. The continuous process of bone remodeling is achieved by the finely balanced activity of osteoblasts in bone synthesis and osteoclasts in bone resorption. Both osteoblasts and osteoclasts express functional Epors, but the underlying mechanism of Epo‐Epor signaling in bone homeostasis is incompletely understood. Two recent publications have provided new insights into the contribution of endogenous Epo to bone homeostasis. Suresh et al examined Epo‐Epor signaling in osteoblasts in bone formation in mice and Deshet‐Unger et al investigated osteoclastogenesis arising from transdifferentiation of B cells. Both groups also studied bone loss in mice caused by exogenous human recombinant EPO‐stimulated erythropoiesis. They found that either deletion of Epor in osteoblasts or conditional knockdown of Epor in B cells attenuates EPO‐driven bone loss. These findings have direct clinical implications because patients on long‐term treatment for anemia may have an increased risk of bone fractures. Phase 3 trials of small molecule inhibitors of the PHD enzymes (hypoxia inducible factor‐prolyl hydroxylase inhibitors [HIF‐PHIs]), such as Roxadustat, have shown improved iron metabolism and increased circulating Epo levels in a titratable manner, avoiding the supraphysiologic increases that often accompany intravenous EPO therapy. The new evidence presented by Suresh and Deshet‐Unger and their colleagues on the effects of EPO‐stimulated erythropoiesis on bone homeostasis seems likely to stimulate discussion on the relative merits and safety of EPO and HIF‐PHIs.

## Graphical abstract

The HIF‐PHD‐Epo axis is activated by prolyl hydroxylase inhibitors. Schematic representation of the oxygen‐sensing mechanism that regulates transcription of genes containing a hypoxia response element (HRE). In the presence of oxygen, PHD site‐specifically hydroxylates HIF‐α, targeting it for degradation by VHL. In the absence of oxygen HIF‐1α dimerizes with its stable partner HIF‐1β in the nucleus (also known as ARNT) to facilitate a complex array of gene transcriptional responses to hypoxia through its three isoforms (HIF‐1α, HIF‐2α, and HIF‐3α). The three prolyl hydroxylase paralogues, PHD1, PHD2 and PHD3 are dioxygenases, which utilize molecular oxygen (O_2_) and 2‐oxoglutarate (2‐OG) for HIF‐α hydroxylation. PHD2 is the principal regulator of HIF activity in most cells. In the case of erythropoietin, PHD2 hydroxylates HIF‐2α, whereas glycolytic genes such as PGK are HIF‐1α‐driven. Early HIF‐PHIs (hypoxia‐inducible factor prolyl hydroxylase inhibitors), such as Roxadustat, were designed to incorporate a side chain structurally analogous to 2‐OG.

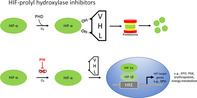

**Significance statement**

Recombinant erythropoietin (EPO) is widely used in clinical practice to treat anemia in chronic kidney disease and other disorders. Long‐term use can lead to reduction in bone mass. The present study highlights two recent publications that have defined new roles for endogenous erythropoietin signaling in bone remodeling. The evidence presented on the role of osteoblast and osteoclast activity during EPO‐stimulated erythropoietic response seems likely to stimulate the debate on the relative efficacy and safety of EPO vs hypoxia inducible factor‐prolyl hydroxylase inhibitors (HIF‐PHIs) for patients who require long‐term treatment for anemia.

## 1 INTRODUCTION

Bone homeostasis is a highly organized process that requires the activity of multiple cells to coordinate bone formation and resorption. Osteoblasts, derived from mesenchymal stem cells, are responsible for bone matrix synthesis and mineralization, whereas osteoclasts derived from myeloid progenitor cells, are responsible for bone resorption. This continuous remodeling is finely balanced and is maintained by the combined action of growth factors and cytokines in the hypoxic bone marrow environment, and systemic factors such as calcitonin and estrogens.^1^, ^2^

During embryogenesis blood precursors migrate and colonize spaces created in the primitive bone marrow.^3^ This close physical association provides the basis for the coordination between bone homeostasis and erythropoiesis in the bone marrow cavity that is maintained throughout adult life. The bone marrow produces 200 billion red blood cells per day, regulated by the classical negative feedback loop in which tissue oxygenation in the kidney controls the production of the hormone erythropoietin (Epo). Circulating Epo binds to the Epo receptor (Epor) on erythroid progenitors in the bone marrow and promotes their proliferation and differentiation to mature red cells. Peritubular interstitial cells, the source of Epo in the kidney^4^, ^5^ utilize the hypoxia inducible factor (HIF) pathway to regulate Epo production in an oxygen‐sensitive manner.

Over the past two decades, the versatility of Epo as a critical regulator of multiple nonerythroid cells has been confirmed, and the role of Epo in bone homeostasis has been repeatedly studied but the underlying mechanism remains incompletely understood.^6^, ^7^, ^8^, ^9^ Functional Epor is expressed on bone marrow stromal cells,^9^ preosteoclasts,^8^ osteoblasts,^8^, ^10^, ^11^, ^12^, ^13^ and immune cells.^14^ The observations that osteoblasts have a role in the regulation of multiple hematopoietic lineages in the bone marrow,^15^ and also produce Epo^16^ point to the presence of an intricate, highly synchronized network of cells and paracrine factors that operates in the hypoxic environment of the bone marrow.

Recombinant human erythropoietin (herein designated EPO) is widely used in clinical practice to treat anemia in patients with chronic kidney disease (CKD) and other disorders. However, EPO treatment initiates additional confounding effects that include the reduction of bone mass in mice^7^, ^8^, ^10^, ^17^, ^18^ and an increased risk of fractures found to be associated with high Epo levels in humans.^19^, ^20^ It is debatable whether EPO therapy induces bone loss through a direct impact on osteoblasts and osteoclasts, or through an indirect effect linked to increased erythropoiesis.

Two recent publications have provided new insights into the contribution of endogenous Epo to bone homeostasis, and the mechanism of bone loss during EPO‐stimulated erythropoiesis. Suresh et al^21^ examined the role of Epo‐Epor signaling in osteoblasts in bone formation in mice, while Deshet‐Unger et al^22^ investigated the controversial topic of osteoclastogenesis arising from the transdifferentiation of B cells and the influence of EPO on this process. Both groups also studied the mechanism of bone loss caused by exogenous EPO. Intriguingly, they found that either deletion of Epor in osteoblasts or conditional knockdown (cKD) of Epor in B cells attenuates EPO‐driven bone loss.

### 1.1 The role of endogenous Epo in bone homeostasis

**FIGURE 1:**
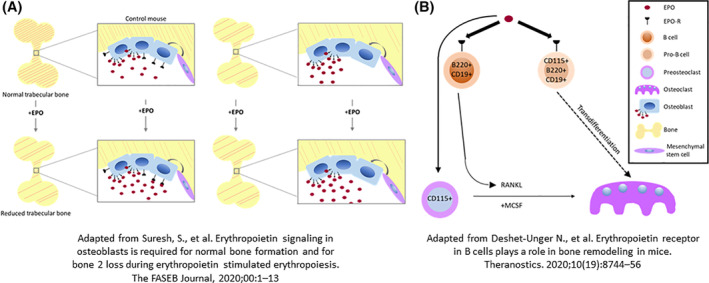
The role of Epo‐Epor signaling in bone development and remodeling. A, Epor expression in osteoblasts is required for bone loss in EPO‐stimulated erythropoiesis. In addition to its cardinal effect of enhancing erythropoiesis, recombinant human erythropoietin (EPO) has nonerythroid effects that include a reduction of bone mass in patients undergoing treatment for anemia. Osteoblasts synthesize bone, express functional Epor, and also produce Epo. Suresh et al found that EPO directly stimulates osteoblasts to decrease bone mass. Transgenic mice (Tg) with osteoblastic‐specific deletion of Epor exhibit reduced bone. EPO administration (1200 U/kg) for 10 days reduced trabecular bone in control mice, but not in Tg mice. B, Epor expression in B cells contributes to osteoclastogenesis and bone remodeling. In common with osteoblasts, osteoclasts, preosteoclasts, and some B and Pro‐B cells express functional Epor. Deshet‐Unger et al present evidence for an alternative B cell‐derived osteoclastogenic pathway in which EPO treatment increased the osteoclastic factor RANKL, induced Pro‐B cell transdifferentiation to osteoclasts, and increased the number of B cell‐derived preosteoclasts. Mice with conditional knockdown in the B cell lineage displayed higher cortical and trabecular bone loss

Suresh and colleagues generated a new transgenic mouse (Tg), in which the *Epor* was deleted in osteoblasts by crossing *Osteocalcin (Bglap)‐Cre* mice with *EpoR* ^floxp/floxp^ mice in a C57BL/6 background.^23^ The authors confirmed that the *Osteocalcin‐Cre*‐mediated floxed recombination occurred specifically in osteoblasts, and not in nonskeletal tissues such as spleen or white adipose tissue where high levels of *Epor* are observed. In Tg mice Epo‐Epor signaling is specifically ablated in mature osteoblasts (Figure 1A).

They found that Epor signaling in mature osteoblasts related to endogenous Epo is critical for bone development in both male and female mice, and that defective bone development in Tg mice increases with age. Compared with their respective littermates, trabecular bone volume fraction in Tg mice was substantially reduced in both genders. In cortical bone, only female Tg mice showed a reduction in cortical bone density, but cortical thickness and cortical bone mineral density were unchanged. Thus, Epo‐Epor signaling in osteoblasts is essential for bone development, and the role of Epo in the maintenance of trabecular bone is more pronounced than in cortical bone.

Alkaline phosphatase (Alp) activity is crucial for the mineralization of bone and its presence is considered indicative of osteoblast cells. Consistent with the in vivo observation that targeted deletion of *Epor* in osteoblasts reduces bone volume, in vitro osteogenic cultures from Tg mice showed decreased enzymatic activity of Alp, indicating a reduction in osteoblast differentiation potential. Taken together, *Epor* deletion in osteoblasts impairs their differentiation in vitro and reduces trabecular bone in both genders. These observations demonstrate that Epo signaling regulates osteoblasts, thereby defining a novel role for Epo as an essential regulator of bone homeostasis.

Lineage commitment and differentiation of BMSCs and skeletal cells depends upon the interplay of transcription factors and signaling molecules.^9^ Several cytokines present in the bone marrow niche also preferentially regulate BMSC/skeletal stem cells (SSC) differentiation.^24^ Epo is known to promote MSC proliferation while maintaining their multilineage potential.^25^, ^26^, ^27^ MSCs have the potential to differentiate into osseous, cartilaginous and adipose tissue lineages. The effect of Epo on osteogenic differentiation of MSCs has recently been reviewed by Zubareva et al.^28^ The current consensus is that Epo triggers differentiation of MSCs into osteoblasts in vitro,^11^, ^29^, ^30^ mediated through nonhematopoietic receptors and multiple intracellular signaling pathways.^13^, ^31^

### 1.2 The effect of exogenous EPO on bone homeostasis

There is clear evidence of trabecular bone reduction in mice administered with low or high doses of EPO^7^, ^8^, ^9^ and in the Tg6 transgenic mouse model in which human Epo is overexpressed.^8^ Similarly, Suresh and colleagues found that administration of EPO for 10 days reduced trabecular bone in control mice, but not in Tg mice with *Epor* deletion, indicating that Epor signaling in osteoblasts contributes to bone loss. EPO treatment raised hematocrits to similar levels in both control and Tg mice.

Interestingly, Tg mice treated with EPO displayed a sexually dimorphic response. In male mice, a trend for reduced trabecular bone was not as pronounced as the bone loss in wild‐type mice, suggesting that factors other than the EPO response in osteoblasts mediate bone reduction. In contrast, administration of EPO to female Tg mice did not cause a reduction in trabecular bone, indicating that bone reduction in response to EPO in females is either fully regulated by Epo signaling in osteoblasts, or that a protective mechanism exists specifically in females to protect against EPO‐induced bone loss.

Earlier work had shown that the addition of EPO to human osteoblast cultures induces the canonical JAK2/STAT3 signaling pathway indicating active Epo‐Epor signaling in osteoblasts.^12^ Suresh and colleagues now report that osteoblasts in primary osteogenic cultures from Tg mice show reduced differentiation potential and reduced ALP activity, consistent with the reduction in bone volume caused by targeted deletion of Epor in osteoblasts, and reminiscent of mice with attenuated osteoblast Hif‐1/Hif‐2 signaling previously reported.^16^

Further insight into the genotype: phenotype correlations may be gained by study of Epor knockout in bone marrow stromal cells (BMSCs). To date there are no published reports with Epor knockdown specifically in BMSCs, but one study investigated ΔEpoR_E_ mice, in which Epor expression is restricted to the erythroid lineage.^9^ These mice exhibit reduced trabecular bone, increased bone marrow adipocytes, and decreased bone morphogenic protein 2 driven ectopic bone formation. EPO‐treated ΔEpoR_E_ mice attained hematocrits comparable to wild‐type mice, and without further bone reduction, suggesting that bone reduction with EPO treatment is associated with a nonerythropoietic Epo response. This is consistent with the findings of the two new publications, that Epo also exerts its effects through osteoblasts and osteoclasts.

Transplantation of bone marrow stromal cells from wild‐type, ΔEpoR_E_, and Tg6 mice which chronically overexpress human *Epo*, was used to assess the development into a bone/marrow organ in immunodeficient mice.^9^ Analogous to endogenous bone formation in vivo, Tg6 bone marrow cells displayed reduced differentiation to bone and adipocytes, indicating that high EPO inhibits osteogenesis and adipogenesis, while ΔEpoR_E_ bone marrow cells formed ectopic bones with reduced trabecular regions and increased adipocytes, indicating that loss of Epo signaling favors adipogenesis at the expense of osteogenesis. Consequently, endogenous Epo signaling regulates bone marrow stromal cell fate, and aberrant Epo levels result in their impaired differentiation.

### 1.3 Effect of EPO on bone marrow B‐cells

Osteoclasts arise from myeloid progenitor cells such as monocytes and follow clear differentiation pathways. Deshet‐Unger and colleagues probed the hypothesis that osteoclasts also arise from bone marrow B cells.^22^ They found that EPO enhances the ability of B cells to transdifferentiate into functional osteoclasts by upregulating the expression of known osteoclastogenic molecules such as RANKL.^22^, ^32^, ^33^

They investigated B cell‐derived osteoclastogenesis using histological and lineage tracing methods. To determine which cells can transdifferentiate into osteoclasts in vitro, they isolated three populations of B cells from wild‐type mice. When Pro‐B cells (B220^+^CD19^+^CD43^High^IgM^−^), pre‐B cells (B220^+^CD19^+^CD43^Low^IgM^−^), and immature B cells (B220^+^CD19^+^CD43^−^IgM^+^) were subjected to an osteoclastogenic assay, osteoclastogenesis was restricted to a subset of Pro‐B cells expressing CD115 (the CSF1 receptor). Upon stimulation with RANKL and MCSF in culture, these CD115+ Pro‐B cells gave rise to tartrate‐resistant acid phosphatase positive (TRAP+) multinucleated osteoclast‐like cells. Of note, the osteoclasts generated from the pro‐B cell population were smaller than those from the monocyte‐containing cell population (Figure 1B). Remarkably, this phenomenon was not amplified by EPO applied in vitro. However, EPO treatment in vivo increased the number of B cell‐derived preosteoclasts (β3^+^CD115^+^ Pro‐B cells), suggesting that EPO augments B‐cell‐derived osteoclastogenesis also by enriching the pool of B‐cell‐derived osteoclast precursors.

In order to examine the influence of EPO administration on bone marrow B cells in vivo, Deshet‐Unger et al treated normal mice with three injections of 180 IU of EPO and noted a significant increase in the expression of membrane bound RANKL.

To explore the impact of Epo‐Epor signaling in B cells on bone loss, they utilized mice with conditional *Epor* knock‐down (cKD) in the B cell lineage.^34^ Analysis of these cKD femurs by microcomputed tomography revealed an increased cortical and trabecular bone mass when compared with control mice.

To investigate the effect of EPO treatment on B‐cell specific bone loss, cKD and wt mice were given three injections of 180 IU per week for 2 weeks. They found that the cKD mice had attenuated EPO‐driven trabecular bone loss and attained higher hemoglobin levels than EPO‐injected wt control mice.

The exact role of EPO in bone remodeling remains to be determined. Suresh et al found that Epo‐Epor signaling in mature osteoblasts is essential for bone development in mice, and that EPO treatment causes bone loss. Deshet‐Unger et al^22^ demonstrate that EPO treatment in vivo enhances BM B cell expression of RANKL, a pivotal regulator of bone metabolism, suggesting a paracrine effect on osteoclastogenesis. EPO treatment also increased the number of β3+ CD115+ preosteoclasts, implying a physiological rationale for B cell‐derived osteoclastogenesis. Mice with conditional Epor knockdown in the B lineage displayed increased bone mass phenotype in the steady‐state on one hand, and an attenuation of EPO‐driven bone loss on the other, highlighting the central role of the Epo‐Epor signaling pathway in B cells, and the potential involvement of Epo in the modulation of bone remodeling. Earlier studies of the skeletal role of EPO in murine overexpression models established that EPO targets the monocytic lineage by increasing the number of bone marrow monocytes/macrophages, preosteoclasts and mature osteoclasts.^8^ Future studies are needed to clarify the physiological role of Epo in bone remodeling, how this is perturbed by EPO treatment, and to define the relative importance of the cell lineages involved.

## 2 THE HIF PATHWAY IN BONE HOMEOSTASIS AND HEMATOPOIESIS

**FIGURE 2:**
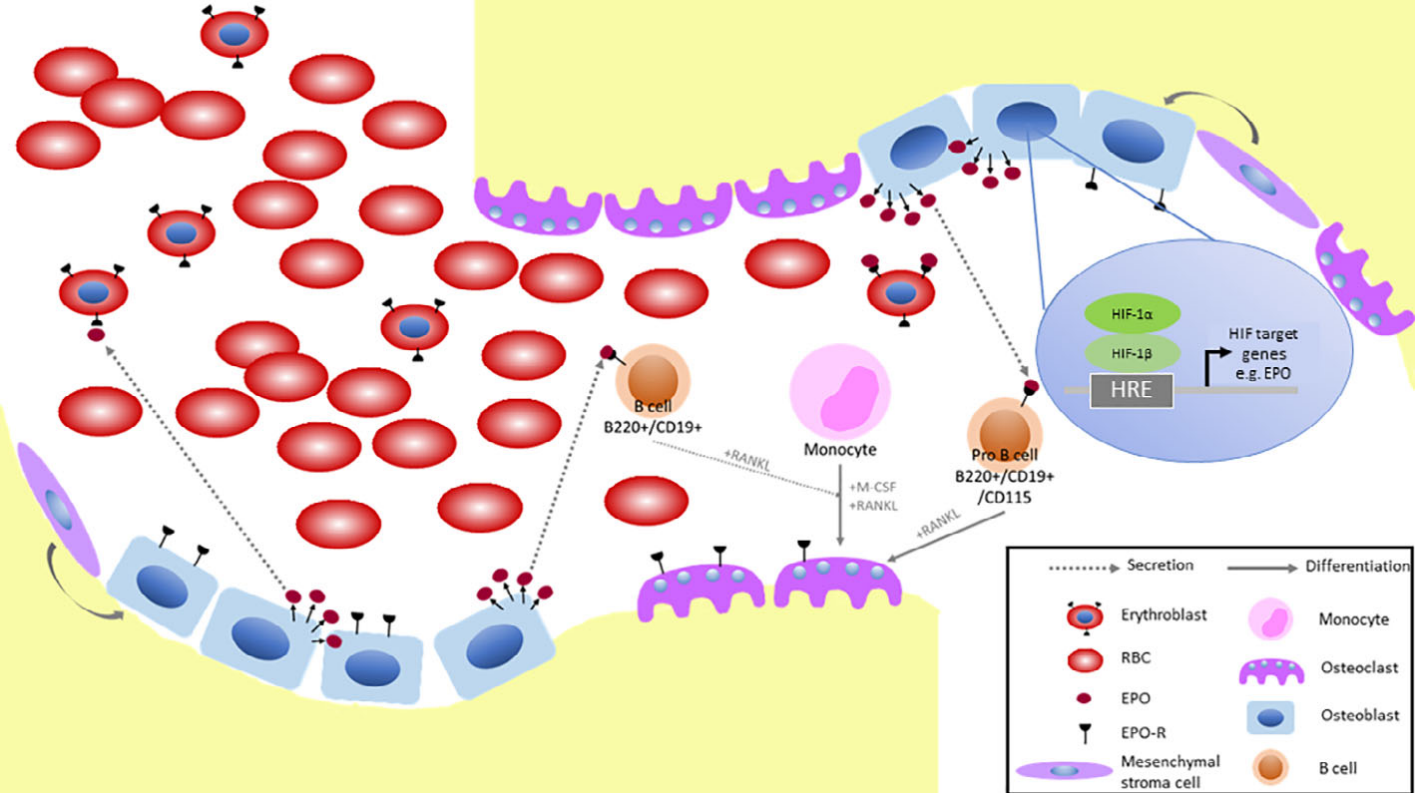
Bone homeostasis and hematopoiesis in the bone marrow. The hypoxic state of the bone marrow provides a supportive environment for hematopoiesis and bone development and resorption. Hematopoietic stem cells (HSCs) give rise to erythroid, myeloid and lymphoid lineages from which respectively erythroblasts, monocytes and B cells are derived. Osteoclasts arise from myeloid progenitors such as monocytes. Epo is critical for erythropoiesis and bone development. Both circulating and osteoblast‐derived Epo are present in the marrow and can activate a wide array of cells involved in bone homeostasis and hematopoiesis

The HIF pathway plays a key role in the ongoing parallel processes of bone homeostasis and hematopoiesis in the bone marrow, a hypoxic tissue with a pO_2_ of approximately 10 to 30 mmHg^35^ (Figure 2). Under these conditions HIF upregulates a wide range of cellular functions including glycolysis and erythropoiesis.^36^, ^37^ HIF is a dimeric complex composed of an α subunit (either HIF‐1α, HIF‐2α, or HIF‐3α) and a β subunit (also known as ARNT), that activate hypoxia response element (HRE) gene transcription in mammalian cells.^38^, ^39^ In the presence of oxygen, a group of dioxygenases known as Prolyl Hydroxylase Domain proteins (PHDs) hydroxylate HIF‐α, targeting it for degradation by the von Hippel Lindau tumor suppressor protein. Under hypoxic conditions, such as in the bone marrow, the degradation is attenuated, HIF‐α is stabilized, binds to its stable partner HIF‐β, and induces gene transcription.

Although HIF‐2α expression is more restricted than HIF‐1α, studies of humans and genetically modified mice have indicated that HIF‐2α plays the predominant role in control of erythropoiesis in adult mammals.^40^, ^41^, ^42^, ^43^, ^44^ HIF‐2α is abundantly expressed in the lungs and vasculature.^45^ HIF‐2α is also a key regulator of iron metabolism.^46^

Bone marrow contains two distinct types of stem cells, hematopoietic stem cells that give rise to the complete repertoire of mature blood cells, and mesenchymal stem cells which can give rise to at least three mature populations including osteoblasts, chondrocytes and adipocytes.^47^ Osteoblastic cells are a component of the hematopoietic stem cell (HSC) niche and reside primarily at the endosteal surface where immature hematopoietic cells are preferentially located.^48^ Osteoblast signaling can alter HSC fate, and contribute to the regulation of multiple hematopoietic lineages including the erythroid and B‐lymphocyte lineages.^15^

**FIGURE 3:**
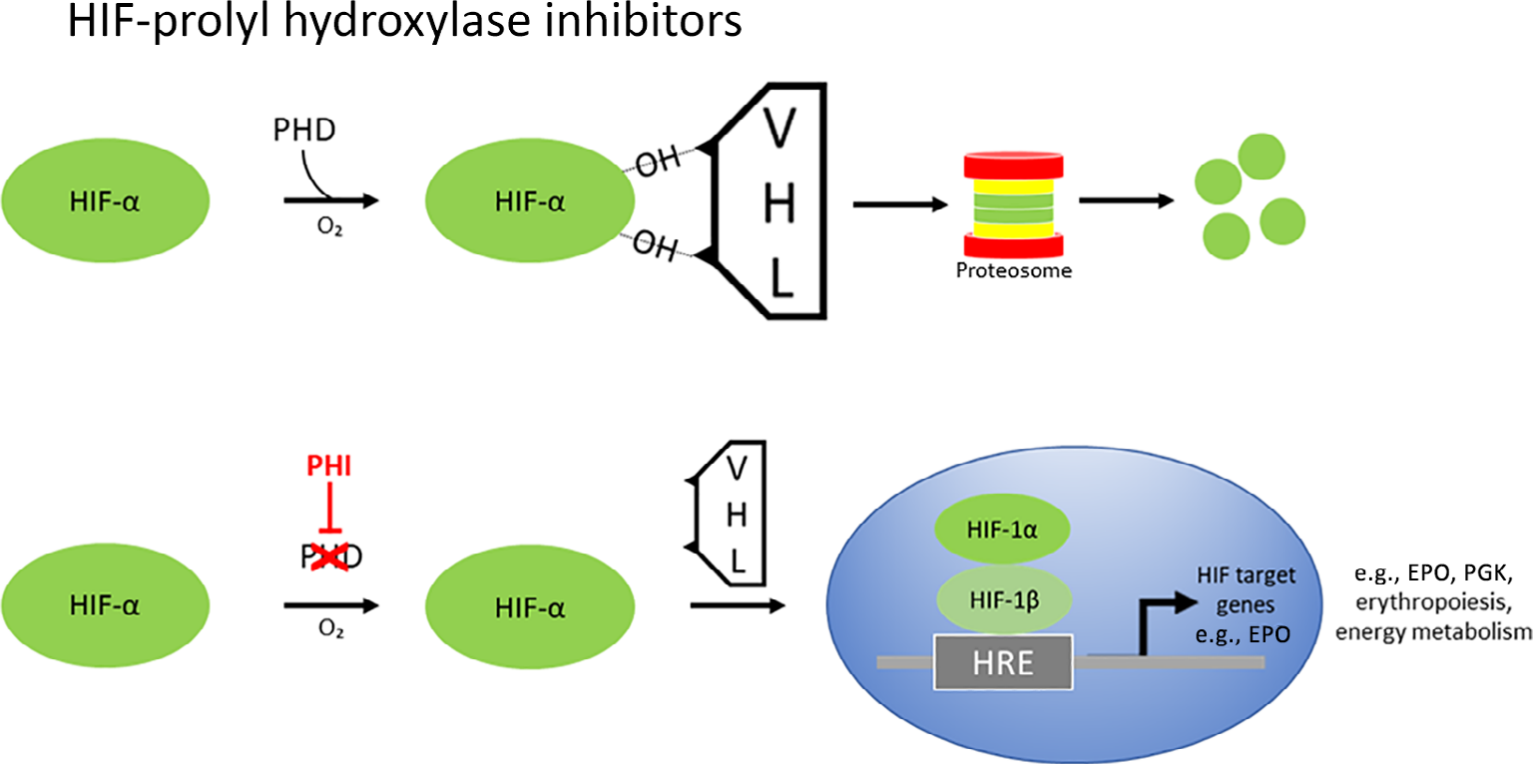
The HIF‐PHD‐Epo axis is activated by prolyl hydroxylase inhibitors. Schematic representation of the oxygen‐sensing mechanism that regulates transcription of genes containing a hypoxia response element (HRE). In the presence of oxygen, PHD site‐specifically hydroxylates HIF‐α, targeting it for degradation by VHL. In the absence of oxygen HIF‐1α dimerizes with its stable partner HIF‐1β in the nucleus (also known as ARNT) to facilitate a complex array of gene transcriptional responses to hypoxia through its three isoforms (HIF‐1α, HIF‐2α and HIF‐3α). The three prolyl hydroxylase paralogues, PHD1, PHD2 and PHD3 are dioxygenases which utilize molecular oxygen (O_2_) and 2‐oxoglutarate (2‐OG) for HIF‐α hydroxylation. PHD2 is the principal regulator of HIF activity in most cells. In the case of erythropoietin, PHD2 hydroxylates HIF‐2α, whereas glycolytic genes such as PGK are HIF‐1α‐driven. Early HIF‐PHIs (hypoxia‐inducible factor prolyl hydroxylase inhibitors), such as Roxadustat, were designed to incorporate a side chain structurally analogous to 2‐oxoglutarate

Osteoblasts, in common with kidney peritubular interstitial cells regulate Epo production in an oxygen sensitive manner.^16^, ^49^ Under normoxic conditions, PHD2 site‐specifically hydroxylates the labile α subunit of HIF‐2α, targeting it for VHL‐mediated proteasomal degradation.^50^, ^51^, ^52^ Under hypoxic conditions, HIF‐2α is stabilized, binds to its stable partner HIF‐β, and induces transcriptional activation of *Epo*,^53^, ^54^, ^55^ see Figure 3.

HIF signaling in osteoblasts is crucial for bone formation and homeostasis. Conditional ablation of Hif‐1 and Hif‐2 causes a reduction in bone volume, but Hif‐1 and Hif‐2 stabilization leads to an increase in bone mass.^49^

Rankin and colleagues^16^ reported that OSX‐VHL mice, which are deficient in Vhl in osteoblasts, thus causing overstabilization of both Hif‐1 and Hif‐2, exhibit disproportionate accumulation of trabecular bone in the long bones, accompanied by a significant increase in circulating red blood cells and decrease in lymphocytes. Furthermore, investigation of OSX‐HIF‐2 mice (defective in Hif‐2), but not OSX‐HIF‐1 mice (defective in Hif‐1), indicated that augmented Hif‐2 signaling in osteoprogenitor cells led to the development of erythrocytosis. Overall, this study confirmed that osteoblastic Hif affects bone formation, and revealed an unexpected role for osteoblasts in the production of Epo and modulation of erythropoiesis.

## 3 CLINICAL INSIGHTS OF EPO‐EPOR SIGNALING

In addition to the evidence for Epo‐mediated bone remodeling obtained from murine models, results from clinical studies point to a functional link between erythropoiesis and bone homeostasis.^19^ Patients with chronic hemolytic anemias such as thalassemia suffer from low bone mass, fractures, and bone pain.^56^ A high proportion of patients that suffer sickle cell anemia also have low bone density.^57^ In chronic hemolytic anemia and in polycythemia there is a continual Epo‐induced drive on the marrow to produce red blood cells.

Anemia is prevalent in the elderly and is often associated with increased risk for bone fractures and low bone mineral density.^58^, ^59^ Circulating Epo levels are low, but treatment with recombinant Epo carries the risk of bone loss.^19^, ^20^ This illustrates the bidirectional effects of recombinant EPO therapy.

Taken together the clinical studies indicate a coordinated regulation of erythropoiesis and bone homeostasis in which elevated Epo signaling enhances red blood cell production at the expense of osteogenesis. They therefore mirror the two preclinical models under discussion, which clearly show that Epo‐Epor signaling is important for erythropoiesis and bone remodeling, and that EPO‐stimulated erythropoiesis causes bone loss.

With regard to the bone loss encountered in patients on long‐term recombinant EPO therapy,^19^, ^20^ an important question is whether small molecule inhibitors of the PHD enzymes, collectively known as HIF‐PHIs (prolyl hydroxylase inhibitors), which result in increased endogenous Epo production, can provide a better approach to stimulating erythropoiesis than EPO, without the attendant bone loss caused by EPO.

Over the past 30 years recombinant human EPO and other erythropoiesis stimulating agents (ESAs) have been used successfully to treat anemia in millions of patients, predominantly in those with CKD. Stimulation of erythropoietic activity increases the demand for iron in the marrow, so the current management of patients with advanced CKD consists of injectable EPO supplemented by injectable or oral iron. Injectable EPO causes supraphysiological levels of circulating Epo, that often accompany intravenous EPO therapy.^60^ HIF‐PHIs enhance HIF signaling and offer the advantage of stimulating red blood cell production and simultaneously increasing the availability of iron to meet the demands of the augmented erythropoiesis. Management of patients is simplified because HIF‐PHIs are taken orally, and their dose can be readily adjusted.

HIF‐PHIs improve iron uptake and utilization by increasing the transcription of a group of enzymes regulated by HIF.^46^ In the gut, iron uptake is promoted by increased transcription of two HIF‐2 regulated genes, *divalent metal transporter 1* (*DMT1*), and *duodenal cytochrome b* (*DCYTB*). DMT1 transports iron into the cytoplasm of the cells and duodenal cytochrome b (DCYTB) reduces iron from its ferric (Fe^3+^) to its ferrous (Fe^2+^) form, permitting its uptake into intestinal cells via DMT1. Furthermore, HIF regulates the genes that encode the iron transporter transferrin, the transferrin receptor, ceruloplasmin which is involved in iron transport, heme oxygenase 1 which enables recycling of iron from phagocytosed red blood cells, and ferroportin, the only known cellular iron exporter.^46^

HIF‐PHIs can provide a more physiological approach for treating anemia than recombinant EPO therapy by maintaining circulating Epo within the physiologic range. They circumvent supraphysiologic increases in Epo levels which has been proposed as a factor in the increased risk of cardiovascular events associated with EPO therapy in advanced CKD.^46^

There are currently more than 15 phase 3 clinical trials worldwide assessing the efficacy and safety of Roxadustat, the first small molecule HIF‐PHI, in CKD patients with anemia.^61^ Roxadustat, is efficacious in increasing endogenous Epo levels comparable to those in the therapeutic range, in a titratable manner. In addition, Roxadustat improves iron metabolism, irrespective of the inflammatory state of the CKD patients, by increasing intestinal iron absorption and decreasing hepcidin production.^61^

Currently chemical ligands which specifically modulate HIF‐2 stability by binding directly to the PAS‐B pocket are being explored, and some first‐in‐class agonists of HIF‐2α have been identified.^62^ Interestingly, other ligands which bind to the same pocket act as antagonists of HIF‐2a stability and may potentially find application in some cancers driven by enhanced HIF‐2 activity. It will be of interest to learn whether some of these small molecules that specifically enhance HIF‐2α function can replace recombinant EPO in the long‐term treatment of anemia. Furthermore, some of the novel small molecules and the clinically approved HIF‐PHDs may find clinical application in the treatment of disorders such as vascular insufficiency and airway degeneration.^63^, ^64^

## 4 CONCLUSION

In summary the new publications from Suresh et al and Deshet‐Unger et al underscore the importance of Epo‐Epor signaling in the complementary relationship between erythropoiesis and bone homeostasis. They define the noncanonical function of Epo in bone homeostasis and demonstrate that Epo signaling in osteoblasts and osteoclasts is essential for bone development and remodeling that occur in the hypoxic environment of the bone marrow. The evidence presented on the role of osteoblasts and osteoclasts activity during EPO‐stimulated erythropoietic response seems likely to further fuel the debate on the relative efficacy and safety of EPO vs HIF‐PHIs for patients who require long‐term treatment for anemia. HIF‐PHIs may provide a means to selectively increase erythropoiesis while maintaining bone condition.^65^

## CONFLICT OF INTEREST

The authors declared no potential conflicts of interest.

## AUTHOR CONTRIBUTIONS

K.M.L., T.R.L.: wrote the manuscript, designed the figures, conceptualization, read and approved the final manuscript; K.I.M.: critically reviewed the manuscript, conceptualization, read and approved the final manuscript.

## Data Availability

Data sharing is not applicable to this article as no new data were created or analyzed in this study.
